# Urlaubssouvenir

**DOI:** 10.1007/s00105-023-05226-2

**Published:** 2023-09-11

**Authors:** Michael Fink, Verena Ahlgrimm-Siess, Rainer Hofmann-Wellenhof

**Affiliations:** 1 Universitätsklinik für Dermatologie und Venerologie, Medizinische Universität Graz, Auenbruggerplatz 8, 8010 Graz, Österreich; 2 Universitätsklinik für Dermatologie und Allergologie, Paracelsus Medizinische Privatuniversität Salzburg, Salzburg, Österreich

## Anamnese

Eine 25-jährige Patientin wurde mit Juckreiz und Hautveränderungen an der linken Brust in unserer Ambulanz für allgemeine Dermatologie vorstellig. Der Juckreiz und die Hautveränderungen bestanden bereits seit ca. 4 Wochen und waren während eines 5‑wöchigen Asienurlaubs erstmalig aufgetreten. Sowohl die Anwendung eines lokalen Steroids als auch die Kombination eines lokalen Steroids mit einem lokalen Antibiotikum hatten zu keiner Besserung geführt.

## Klinik

**Figure 1:**
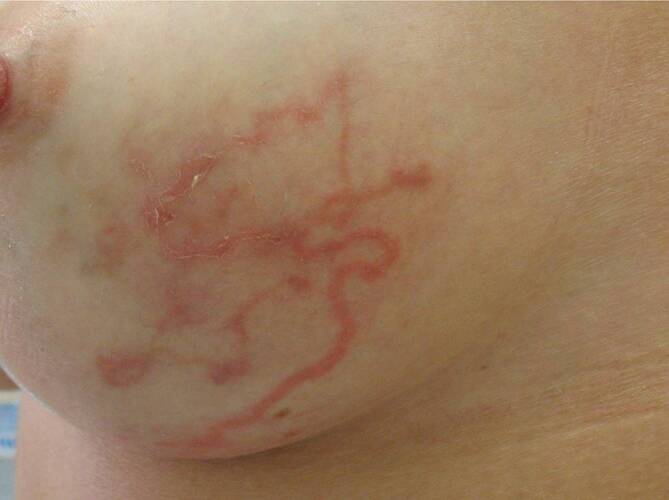

Klinisch zeigte die Patientin erythematöse, girlandenförmige Striae mit geringer lamellöser Schuppung und einzelne kleine Papeln im Bereich der linken Brust (Abb. 1).

## Dermatoskopie

**Figure 2:**
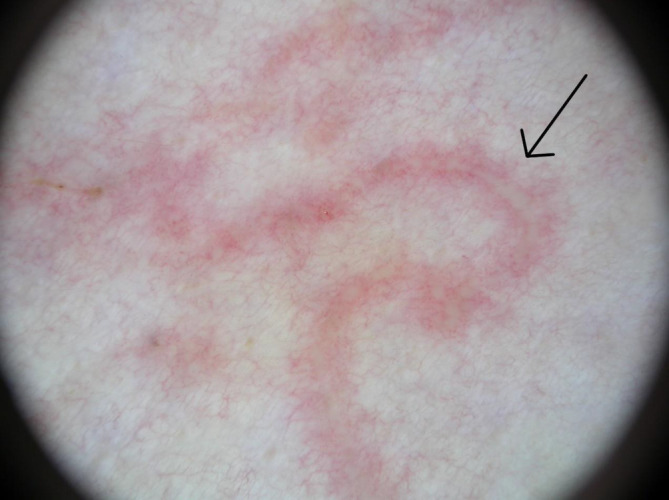

**Figure 3:**
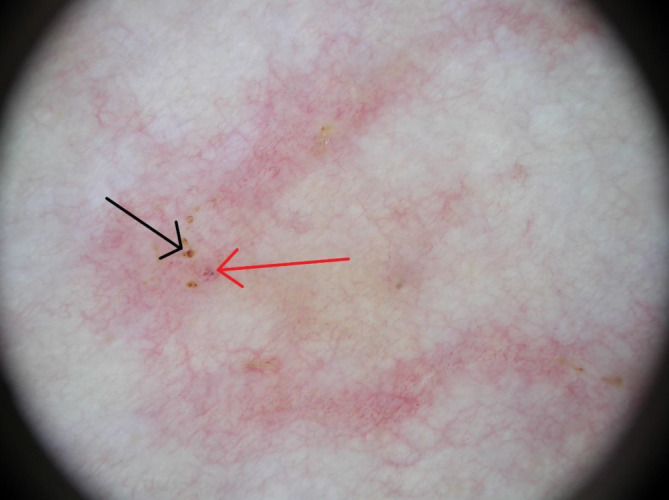

Die Dermatoskopie zeigte im Bereich der klinischen Veränderungen gewundene, streifige Erytheme, in denen fokal gelb-weißliche, lineäre und rundliche Strukturen zu erkennen waren (Abb. 2, Pfeil). Zudem waren zahlreiche rote und vereinzelt livide Punkte (Abb. 3, roter Pfeil) sowie hell- bis dunkelbraune Krusten (Abb. 3, schwarzer Pfeil) zu sehen.

## Verlauf und Therapie

Aufgrund des klinischen Bildes, der charakteristischen Veränderungen in der Dermatoskopie und der Vorgeschichte eines Auslandaufenthaltes wurde zusammenschauend die Diagnose einer kutanen Larva migrans gestellt. Der Patientin wurde systemisches Albendazol und eine Lokaltherapie mit Mometasonfuroat verordnet. Die Patientin wurde jedoch 14 Tage später wegen persistierenden Juckreizes und fehlender Abheilung der Hautveränderungen erneut vorstellig; Albendazol war nicht eingenommen worden und die Lokaltherapie alleine erneut nicht ausreichend. Daraufhin wurden der Patientin nochmals Albendazol sowie eine symptomatische Lokaltherapie verschrieben. Darunter kam es schließlich zur Abheilung der Beschwerden.

## Diskussion

Die kutane Larva migrans ist eine der häufigsten Hauterkrankungen von Reiserückkehrern aus tropischen und subtropischen Gebieten, insbesondere aus Lateinamerika, Afrika und Südostasien [1]. Hervorgerufen wird diese Hauterkrankung durch eine Vielzahl von Hakenwürmern, wobei v. a. *Ancylostoma braziliense, Ancylostoma canium* und *Uncinaria stenocephala* ursächlich sind [2]. Diese Würmer legen Eier im Darm ihres Wirts (meist Hunde und Katzen) und werden in weiterer Folge mit ausgeschieden [2]. Unter optimalen Bedingungen (Wärme, Feuchtigkeit, Schatten und nach Aufnahme von Bodenbakterien) reifen sie zunächst zu nichtinfektiösen und danach zu infektiösen wurmförmigen Larven heran [2]. Die Larven gelangen über direkten Kontakt in die Haut der Menschen, beispielsweise wenn diese barfuß am Strand entlanggehen [1].

Klinisch präsentiert sich die kutane Larva migrans meist mit rötlich-braunen Papeln, welche die Eintrittspforte der Larve darstellen, und den pathognomonischen, erythematös-serpiginösen Striae [1].

Die kutane Larva migrans findet man v. a. an jenen Körperstellen, die mit dem mit Larven kontaminierten Boden in direkten Kontakt kommen, also vorwiegend an Füßen, Rücken und Beinen [1]. Bei unserer Patientin war die Lokalisation an der Mamma eher ungewöhnlich.

Zusätzlich zur klinischen Untersuchung kann die Dermatoskopie in der nichtinvasiven Diagnosesicherung hilfreich sein. Typisch sind, wie auch bei unserer Patientin vorliegend, weiß-gelbliche, lineäre und fokal ovoide Strukturen, die erweiterten Lymphgefäßen entsprechen, in denen sich die Larve fortbewegt [5]. Zahlreiche rote und violette Punkte, die mit punktförmigen Gefäßen korrelieren, sind aufgrund der begleitenden Inflammation erkennbar [3, 4]. In unserem Fall waren zudem braune Krusten zu sehen, die bei einer begleitenden spongiotischen Dermatitis auftreten können [4].

Die Diagnose einer kutanen Larva migrans ist bei Vorliegen des typischen klinischen Bildes und einer passenden Reiseanamnese auch für Nicht-Dermatologen naheliegend. Die Dermatoskopie ist hilfreich, um die diagnostische Sicherheit vor Therapie – v. a. bei klinisch nicht gänzlich eindeutigem Befund – weiter zu erhöhen.

## References

[1] Leung AKC, Barankin B, Hon KLE (2017). Cutaneous larva Migrans. Recent Pat Inflamm Allergy Drug Discov.

[2] Brenner MA, Patel MB (2003). Cutaneous larva migrans: the creeping eruption. Cutis.

[3] Zalaudek I, Giacomel J, Cabo H, Di Stefani A, Ferrara G (2008). Entodermoscopy: a new tool for diagnosing skin infections and infestations. Dermatology.

[4] Rather S, Aslam A, Younus F (2022). Dermoscopy before and after treatment of cutaneous larva migrans: through the dermoscope. Indian Dermatol Online J.

[5] Sandhu S, Bhatnagar A, Suhag D (2023). Dermoscopy of cutaneous larva migrans. Indian J Dermatol Venereol Leprol.

